# Psychological Interventions in Patients with Physical Pain: A Focus on Catastrophizing and Resilience—A Systematic Review

**DOI:** 10.3390/healthcare13060581

**Published:** 2025-03-07

**Authors:** Adriana Leccese, Melania Severo, Antonio Ventriglio, Serena Petrocchi, Pierpaolo Limone, Annamaria Petito

**Affiliations:** 1 Department of Humanistic Studies, University of Foggia, 71122 Foggia, Italy; 2 Department of Clinical and Experimental Medicine, University of Foggia, 71122 Foggia, Italy; 3 Faculty of Biomedical Sciences, Institute of Family Medicine, Università Della Svizzera italiana, 6900 Lugano, Switzerland; 4 Department of Psychology and Education, Università Telematica Pegaso, 80143 Napoli, Italy

**Keywords:** physical pain, catastrophizing thinking, resilience, medical setting

## Abstract

**Background**: Cognitive processes play a crucial role in the perception of pain. Catastrophizing, defined as a tendency to focus on a painful experience or amplify its unpleasantness, even in an anticipated form, might increase patients’ negative expectations and feelings of helplessness. In fact, high levels of pain catastrophizing are associated with a reduction in resilient behaviors among patients with physical pain. The objective of the present study is to investigate the employment of psychological and psychotherapeutic interventions in patients reporting pain, focusing on their improvements in terms of resilience and catastrophizing. **Methods**: This review was conducted following the PRISMA guidelines using three databases including articles published from 2006 to February 2024: PubMed, Scopus, and Web of Science. The search strategy was based on the PIO (Population, Intervention, Outcome) scheme. Following the Cochrane recommendations, quality assessment was performed using the Cochrane Risk of Bias tool (RoB 2.0) for randomized studies and the Cochrane Risk of Bias for NonRandomized Studies (ROBINS-I). We included all English language studies reporting on psychological interventions in the context of pain management and related catastrophic thinking and resilience behaviors. Reviews, book chapters, editorials, conference abstracts and notes, observational studies, and non-English studies were excluded. Two independent authors were involved in the screening and data extraction phase. **Results**: A total of 10 studies were finally selected. The selected studies included five randomized controlled trials (RCTs); three single group, nonrandomized pilot trials; one single case study; and one nonrandomized pilot trial. The studies were mostly conducted in the United States (n = 7). Overall, the studies reported that psychological interventions in patients with physical pain may improve their psychological health and pain management. Reduced levels of catastrophic thinking and improved psychological resilience were found in subjects undergoing psychological treatments in most selected studies. **Conclusions**: Further studies are needed, above all controlled trials, to confirm the impact of these interventions in improving patients’ emotional and physical health in the long-term management of physical pain, improving their resilience and reducing catastrophic thinking.

## 1. Introduction

Pain is a common reason why patients may seek medical assistance [1]. The International Association for the Study of Pain (IASP) defines chronic pain as “an unpleasant emotional and sensory experience associated with acute or potential tissue damage” [2] (pp. 1976–1982). In general, pain plays an essential survival function because it may indicate the location, type, and intensity of a pathological process, signaling a possible injury or physical discomfort [3]. Over the last few decades, the multi-componential aspects of pain have been analyzed. The sensory component concerns the physical characteristics of pain (e.g., location and intensity); the affective component considers the emotional experience related to pain (e.g., fear); and the evaluative component relates to the subject’s judgment and expectations related to pain [4]. Pain associated with a permanent medical condition that lasts at least three months acquires the connotation of chronicity [5]. According to the current evidence, more than one third of the world population suffers from chronic pain [6]. Pain has been reconsidered with respect to the dualistic ‘mind-body’ view of the biomedical model. According to this perspective, pain is a direct consequence of pathological conditions or organic damage [7]. Thus, it is expected that medical interventions may reduce the damage, and consequently its related pain.

Physical pain may show an independent evolution beyond the type, its severity, and the outcome of the injury [8]. The biopsychosocial perspective posits that biological factors interact with the psychological and social variables of pain to shape the overall outcome [9]. This may explain the intra-individual and inter-individual variability in pain experiences among patients [10]. Also, individual cognitive performances play a role in the different responses to a painful experience. Pain catastrophizing, in particular, is a psychological variable that may modulate the development, perception, and course of pain [11].

With the emergence of cognitivism in the 1960s, in the context of Rational Emotive Behavioral Therapy (REBT), Ellis [12] defined catastrophic thinking as an irrational belief commonly found among people with depressive and anxiety disorders. Within the same diagnostic categories, Beck [13] described catastrophizing as cognitive distortion or automatic negative thinking. Later, Sullivan and colleagues [14] focused their attention on the social character of catastrophizing, defining it as a community-based strategy to obtain support from others. Thus, catastrophizing might be defined as the tendency to focus attention on painful stimuli, negative expectations, and feelings of helplessness, amplifying the unpleasant experience of pain [15]. Specifically, pain catastrophizing is the tendency to amplify the threateningness attributed to a painful event, associated with a feeling of helplessness in relation to pain. It also refers to the difficulty in inhibiting pain-related thoughts during or after the perception of pain, but also in anticipation of it [16]. It has been also interpreted as a multifaceted construct characterized by rumination, magnification, and helplessness [11].

Several authors observed that catastrophizing significantly increases patients’ sensitivity to and worries about pain [16]. Magnification, also described as the difficulty in disengaging from painful rumination and selective attention given to painful sensations, increases the likelihood of using ineffective coping strategies [17] and impeding the use of resilience skills. In fact, focusing only on the threatening elements of pain may reduce the possibility of identifying physical or relational positive resources in the surrounding environment [3].

One aim of psychosocial interventions in pain management is to enhance patients’ ability to explore a broader range of strategies, fostering a more flexible outlook. Observations of individuals’ responses to painful or adverse events suggest that variability in coping abilities can enable some to experience positive emotions despite their painful condition [18]. This concept may include the construct of resilience, from the Latin ‘resilire’ meaning ‘to jump back’, and refers to the ability to cope with significant physical and psychological situations [19] and positively recover from stressful and adverse events. In the psychological field, resilience is defined as the ability to withstand negative events by positively reorganizing one’s life.

Ong and colleagues [20] observed that high levels of resilience support patients’ ability to feel positive emotions, while reducing the catastrophizing components related to the painful experience. More recently, some studies have observed that the acceptance of one’s physical condition and perceived self-efficacy in a sample of patients with chronic pain were inversely associated with catastrophizing a pain experience [21]. Thus, reducing the tendency to catastrophize might require expanding the search for effective coping strategies, encouraging a more flexible view of stressful events and enhancing personal resilience characteristics.

The current pain management models primarily rely on interventions grounded in biomedical principles. However, the evidence supports the effectiveness of psychotherapeutic approaches that aim to reduce catastrophizing and enhance resilience, adopting a biopsychosocial perspective [11]. Research on biopsychosocial models suggests that psychotherapeutic programs can simultaneously improve a person’s quality of life, enhance their psychological well-being, support their physical and functional recovery, and reduce their healthcare utilization and costs [22].

The main objective of the present systematic review was to investigate the documented employment of psychological and psychotherapeutic interventions in patients with physical pain, focusing on the impact of these interventions on patients’ levels of resilience and catastrophizing.

## 2. Materials and Methods

### 2.1. Eligibility Criteria

The PIO (Population, Intervention, Outcome) method, a simplified and more flexible version of PICO (Population, Intervention, Comparator, Outcome), which is useful when the comparator is not present, was used to define the criteria for selecting suitable studies [23,24]. (a) P (Participants): Patients experiencing pain in any clinical or experimental context. No restrictions based on age, sex, or health condition were applied; (b) I (Intervention): Psychological interventions in patients with physical pain. Interventions not directly related to the psychological approach, such as medical, pharmacological, and physiotherapy interventions; (c) O (Outcome): The measurement of catastrophic thinking and resilience using specific validated psychometrics tools. The studies that did not measure catastrophic thinking or resilience as distinct constructs were excluded. Other details are reported in Table 1.

### 2.2. Search Strategy and Study Selection

**Figure 1:**
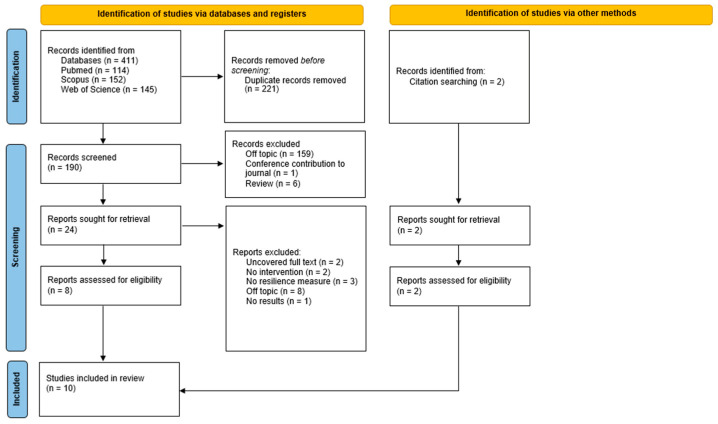
The details of the literature search and the study selection procedure according to the PRISMA flowchart.

A systematic review was conducted according to the principles of the Preferred Reporting Items for Systematic Reviews and Meta-Analyses (PRISMA, Figure 1) guidelines [25,26]. The aim was to summarize the main evidence published in the existing literature.

The protocol was retrospectively registered on PROSPERO, n. CRD42024606274, available at https://www.crd.york.ac.uk/prospero/display_record.php?RecordID=606274 (last accessed 30 November 2024). We acknowledge that post hoc registration may raise concerns about the perceived methodological rigor of this review. To address these concerns, we have implemented measures to minimize potential biases throughout the review process, ensuring the integrity and transparency of our findings. The team of authors consisted of licensed psychotherapists and researchers. A search was conducted on 3 databases (PubMed, Scopus, and Web of Science) and focused on the literature published from 2006 to February 2024. Studies from certain times or locations were not restricted. Considering the limited literature on the topic selected, the same search query was used across all the three databases, combining the keywords ‘resilien*’, ‘pain’, and ‘catastroph*’ with the Boolean operator ‘AND’. Queries were adapted to the specific syntax of each database. The use of asterisks in the query made it possible to expand the search to related words or synonyms. Specifically, resilien* made it possible to include the words resilience, resilient, and resiliently, while catastroph* made it possible to include catastrophizing, catastrophic, catastrophe, catastrophically, catastrophism, catastrophization, and catastrophize. The decision to limit the search to a few keywords was motivated by the need to focus on theoretically defined and specific constructs, such as resilience and catastrophizing, avoiding similar, but not overlapping variables and ensuring consistency with the objectives of the study. For each database, no start date limit was applied. Additional studies were identified by reviewing the reference lists of selected articles. In line with the study’s objectives, articles were included if they met the following criteria: international trials that implemented or evaluated the effectiveness of psychological interventions in the context of pain and studies that specifically measured catastrophic thinking and resilience. This review considered all the relevant literature on the topic published in English. Reviews, book chapters, editorials, conference abstracts, and notes were excluded. Studies that assessed resilience as a component of other variables (e.g., positive emotions, self-efficacy, and acceptance of pain) were also excluded. Studies that explored catastrophic thinking by measuring it as a facet of other variables e.g., negative thinking, rumination, or magnification., were excluded. To ensure consistency, the title, abstract, and full text were analyzed independently by two authors, A.L. and M.S. Any disagreements in the selection of the articles were examined and resolved jointly before proceeding. No significant disagreements emerged regarding the inclusion or exclusion of the articles; however, a third reviewer, A.P., intervened in case of doubts to make a final decision.

### 2.3. Data Extraction and Outcomes

A data extraction table was created to standardize the process (Table A1 and Appendix A). The following information was extracted from each included study and recorded in a data extraction table Microsoft Excel format to ensure consistency across the researchers: article title and author(s), publication date, country of origin, study design, sample size, patients’ gender and age, medical diagnosis related to pain, psychological intervention type, number and modality of intervention session (duration, frequency, individual or group setting, or in-person or online setting), variables evaluated, and clinical outcomes (pre- and post-intervention and other follow-ups if any). The main evaluated outcome(s) was the variation in patients’ resilience and/or catastrophizing thinking levels from baseline to the last follow-up after the interventions measured using validated psychometric instruments. Any other evaluated clinical outcomes considered in the included papers were added to Table A1 even though their analysis was far from the main objective of this systematic review. Finally, through qualitative analysis, the outcomes of the eligible studies are presented in the Results section, first examining the characteristics of the sample, the research design, and the type of psychological interventions implemented. Subsequently, the results on catastrophizing and resilience are reported.

A.L. and M.S. were jointly responsible for identifying the items using databases and eliminating duplicates. Then, throughout the screening phase, the two reviewers worked separately and independently to minimize the Risk of Bias and to achieve an objective assessment of the data examined. In cases of ambiguity or discrepancy, an independent third-party reviewer, A.P., was available to review the matter and make a final decision. The inter-rater agreement was computed with Cohen’s κ = 0.95. The same procedure adopted in the data extraction phase to ensure agreement between the reviewers was also applied in the qualitative summary of the results. This intervention ensured that disputes were resolved impartially, minimizing the possibility of errors due to differing interpretations. In case of disagreement between the first two reviewers, the third reviewer A.P. was responsible for examining the discrepancies and consulting the original material to confirm the correct extraction of the data. Inter-rater agreement computed with Cohen’s κ = 0.92. Finally, each article was reviewed by A.L. and M.S., who extracted the data using a pre-established extraction table. This table included specific information to be collected and how to interpret the data to ensure uniformity and consistency in the approach.

To assess the effect measures of the extracted results, standardized mean differences between the experimental groups or with the control groups were examined (based on the means and standard deviations of each group for pre- and post-assessment scores). In cases where the quantitative outcome data were missing from the studies, we conducted qualitative synthesis, which was deemed sufficient to support meaningful conclusions.

### 2.4. Risk of Bias Assessment

**Figure 2:**
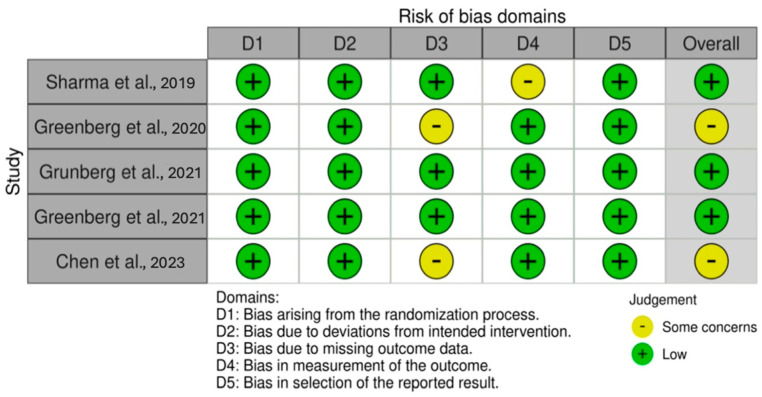
Robvis—Risk of Bias assessment of randomized control trial [29,30,31,32,33].

**Figure 3:**
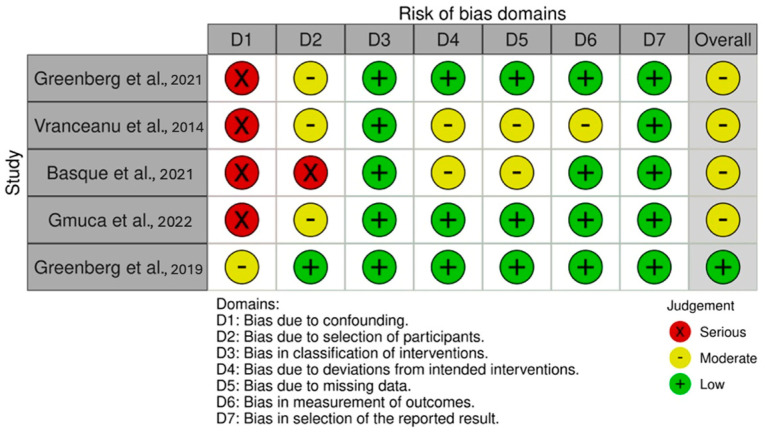
Robvis—Risk of Bias assessment of nonrandomized studies of intervention [34,35,36,37,38].

Consistent with the Cochrane recommendations [27], the criteria used to assess internal validity were identified using robvis [28], a visual tool for assessing Risk of Bias (see Figure 2 and Figure 3) based on the Cochrane Risk of Bias tool (RoB 2.0) for randomized trials and the Cochrane Risk of Bias for NonRandomized Studies (ROBINS-I). With respect to the different domains, the Risk of Bias was assessed as “low”, “high”, or “some concerns” in RoB 2.0 and as “low”, “moderate”, “serious”, or “critical” in ROBINS-I. The characteristics to be evaluated in Rob 2.0 were the following: randomization, deviations from the intended intervention, missing data, outcome measurement, and the selection of reported outcomes. Additionally, the characteristics evaluated for ROBINS-I were confounding factors, participant selection, intervention classification, deviations from the intended intervention, missing data, outcome measurement, and the selection of reported outcomes [28]. The Risk of Bias assessment was carried out independently by A.L. and M.S. who reached a consensus on the definitions for the different domains. Inter-rater agreement was computed with Cohen’s κ = 0.91. Any discrepancy was resolved by A.P. as the third reviewer. Risk of Bias assessment was carried out following the RoB 2.0 and ROBINS-I criteria on an excel templates available on the https://www.riskofbias.info/ website (accessed on 31 January 2025). Subsequently, the excel template was uploaded to the same site, which made it possible to produce a visual representation of the evaluated criteria through the robivis visualization tool [28]. The results of the Risk of Bias assessment are summarized in Figure 2 and Figure 3 through robvis.

### 2.5. Study Selection Process

The flowchart (Figure 1) summarizes the paper selection process. From a total of 411 articles, 221 duplicates were removed. The remaining 190 articles were screened for title and abstract. Of these, 166 were excluded; 159 were off-topic, 6 were review articles, and 1 was a conference paper. In the second phase of screening, 2 articles were excluded due to the unavailability of the full text, 2 did not include a psychological intervention protocol, 3 lacked measures of resilience, 8 were off-topic, and 1 was a study protocol without results. Finally, 2 additional articles were included through a citation search. A systematic review was then conducted on the remaining 10 articles, which were classified by author(s), year, research design, sample, diagnosis, the type of psychological or psychotherapeutic intervention, session characteristics, control group, evaluation measures, and results (Table A1 in Appendix A).

## 3. Results

The selected studies included five randomized controlled trials (RCTs) [29,30,31,32,33], three single group nonrandomized pilot trials [35,36,37], one single case study [34], and one nonrandomized pilot trial [38]. The studies are geographically distributed as follows: one study from Nepal [29], seven studies from the USA [30,31,32,34,35,37,38], one study from China [33], and one study from Canada [36].

### 3.1. Quality Appraisal

Of the five included randomized controlled trials, three were assessed as having a low Risk of Bias, while the remaining two raised concerns due to missing data, highlighting issues with how this limitation was addressed within the studies.

In evaluating the five nonrandomized studies, four were found to have a moderate Risk of Bias due to several factors: the absence of a control group, inadequate procedures used to address potential confounding factors in assessing intervention efficacy, the participant recruitment strategy (e.g., the inclusion of only individuals with internet access or requiring payment to participate), and high dropout rates during the intervention period (Figure 3).

Overall, no deficiencies were identified in the selection of reported outcomes, as the authors presented both significant and nonsignificant results. However, the high dropout rates and the confounding factor bias in the uncontrolled, nonrandomized studies significantly limited the ability to draw definitive conclusions regarding the effectiveness of the interventions. Additionally, substantial differences in research designs were observed among the included studies. Consequently, a narrative synthesis was chosen to qualitatively describe the main outcomes, despite the variability and heterogeneity across the studies.

### 3.2. Participant Characteristics

This research considered a sample of 559 subjects. However, five studies were conducted on the same samples. Specifically, of these five studies, three were conducted on the same sample including 82 patients [30,31,32], and two others included 13 [38] and 4 patients [34], respectively, from the same data collection. The participants were predominantly female (n = 148, 26.48%), with an age ranging from 12 to 79 years old. One study did not specify the gender of participants [31], while one study examined a sample of women only [34]. The reviewed studies showed the heterogeneity of samples regarding diagnosis. Particularly, eight studies examined populations with heterogeneous musculoskeletal pain (neck, head, limbs, shoulder and lumbar areas) [29,30,31,32,33,34,37,38]; one was focused on general chronic pain [36], one on Neurofibromatosis type 1 (NF1), and on Neurofibromatosis type 2 (NF2) or Schwannomatosis [35].

### 3.3. Procedures and Methodologies of the Studies

Assessment conducted in an in-person setting was used in eight studies [29,30,31,32,33,34,35,38]. One study was conducted in an online setting [36]. One study allowed the patients to choose the assessment mode: in-person, via web-based communication, or by telephone [37]. Seven studies included group interventions with numbers of participants ranging from three to sixteen [30,31,32,33,34,35,38]. The number of sessions provided in the interventions was quite homogeneous, with an average number of eight sessions per week. The duration of sessions also ranged from 90 to 150 min. Three studies have included follow-up evaluations at 3 months [32,33,36]. All the studies included at least one assessment of catastrophic thinking and resilience. Nine studies assessed anxiety–depressive symptoms [29,30,31,33,34,35,36,38], and ten studies also included assessment measures of various variables [29,30,31,32,33,34,35,36,37,38]. Only one study used a functional magnetic resonance imaging technique [33]. Finally, four studies did not include the control group [33,34,35,36]. Table A1 in Appendix A provides a summary of the study characteristics.

### 3.4. Type of Interventions

The type of interventions included in this review was rather heterogeneous. Six studies included the Relaxation Response Resiliency Program (3RP) [35]. This program is an outpatient intervention inspired by the principles of mind-body medicine, working on mindfulness and daily practice (teaching healthy lifestyles) for a mind-body balance connection. Its implementation aims to improve coping skills in stressful situations and patients’ quality of life. The sessions were characterized by a common core element based on the elicitation of relaxation response using self-care techniques focused on meditation, guided visualizations, mindfulness, contemplation, and yoga. The program also used tools for cognitive restructuring and the promotion of positive psychology skills (e.g., positive affect, humor, empathy, and giving and receiving help). Five of the other studies based on 3RP showed the results of a program conducted by the same group of U.S. researchers on the use of GetActive With or without Fitbit. GetActive is a mind-body activity program based on the modified Mind-Body Relaxation Response Resiliency Program for pain in group settings directed at chronic musculoskeletal pain [30,31,32,34,38]. This program retained the core components of the mind-body 3RP modified for pain. The final version of the GetActive and GetActive-Fitbit programs included 10 weekly 90 min sessions aimed at acquiring new specific skills to manage pain. These programs included the teaching of mind-body skills focused on activating the relaxation response (e.g., diaphragmatic breathing and body scans) and mindfulness skills (guided meditation), cognitive-behavioral skills specific to pain management (e.g., behavioral reactivation and cognitive restructuring, such as catastrophizing and fear avoidance), and physical restoration skills through a gradual increase in activity, regardless of the pain levels. Only the GetActive-Fitbit version included the gradual increases in activity reinforced by a commercially available digital monitoring device (Fitbit).

Two studies included psychoeducation interventions. Specifically, one used an education intervention focused on pain accompanied by a home program that focuses on improving patients’ understanding of the biological processes underlying pain and strategies to promote recovery [29]. The other psychoeducation intervention was a Self-Compassion psychoeducation online program [36] based on 6 weeks of exercise found on Neff’s psychoeducational website [39]. The intervention included a video defining self-compassion, four self-compassionate writing exercises, guided meditation, and automated emails sent to the participants.

Gmuca and colleagues [37] used the Cognitive-Behavioral Therapy (CBT)-based Promoting Resilience in Stress Management (PRISM) program, an established resilience coaching program for youths with a chronic illness. It aimed to develop resilience and coping skills in four individual sessions and one optional session lasting 30–50 min, either weekly or fortnightly, for a total duration of about 3 months. Sessions 1–4 included stress management, goal setting, cognitive restructuring, and meaning making. The fifth and final session included an optional meeting with the family. Finally, one study used an intervention based on 8-week protocol of Mindfulness-based Stress Reduction (MBSR) developed by Jon Kabat-Zinn and colleagues [40]. Chen and colleagues [33] modified the protocol by adapting it to adults with chronic pain, specifically reducing the duration from 2.5–3 h to 2–2.5 h and eliminating one-day withdrawal for a total duration of 17 h of intervention over 8 weeks. The modified MBSR protocol focused mainly on trauma-informed methods, on dissociating painful sensations, ruminative thoughts and painful sensations, ruminative thoughts, and pain, emphasizing love and kindness toward people feeling distress and compassion.

### 3.5. Resilience and Catastrophizing

Following the interventions, improvements in the overall scores of the psychological variables were observed across all the 10 studies.

Regarding resilience, Sharma and colleagues [29] showed that patients with low back pain did not benefit from the pain education intervention that aimed to normalize the pain experience and provide information on the physiology of pain and the body’s responses [29]. Furthermore, in the same study, this variable remained unchanged following a physiotherapy program. Although no change in resilience skills was observed in this sample of patients with low back pain, a reduction in catastrophizing levels was observed [29]. Compared to the control group undergoing a physiotherapy program, the subjects in the experimental group reported a two-fold reduction in levels of catastrophizing thinking post-intervention. In contrast, the 3RP program by Vranceanu et al. [35] favored, in 16 subjects with the diagnosis of Neurofibromatosis type 1, type 2 or Schwannomatosis, an increase in the scores of resilience, with effect sizes ranging from 0.73 to 1.33. At the same time, a reduction in catastrophic thoughts related to the disease was observed in nine of sixteen participants, while in three subjects, there was no change, and in four, the variable scores worsened. Of the latter, three subjects had undergone surgery during the administration of the psychotherapeutic intervention, an event that may have influenced the outcome of the psychological treatment. The Self-Compassion psychoeducation website by Basque et al. [36] showed an increase in resilience to pain defined as the perceived ability to regulate emotions and thoughts and tenacity in the face of intense or prolonged pain (effect size = 0.55). Additionally, the authors noted an increased acceptance of chronic pain (effect size = 0.43) and a reduction in catastrophizing of anticipated or actual pain (effect size = 0.58), with most outcomes being sustained or further improved at the follow-up.

Five of the studies included in this review reported results consistent with those from previous research conducted by the same group of U.S. researchers using the “GetActive With or Without Fitbit” intervention [30,31,32,34,38]. In an early clinical trial on 22 patients [38], the GetActive group showed significant improvement in pain resilience, with an effect size of 1.11. On the other hand, the participants in the GetActive group with Fitbit showed non-significant improvement in pain resilience. In both the groups, the authors found no improvement in catastrophizing pain. The subsequent developments of this clinical trial included a larger sample of participants (n = 82) showing significant and medium effects in improving pain catastrophizing in both the groups (effect size from 0.43 to 0.72) [30]. Regarding pain resilience, the GetActive group showed significant medium-sized improvements, while the improvements in the GetActive-Fitbit group reported a small effect and did not reach significance. The results from a subsequent study revealed a decrease in pain catastrophizing (effect size from −0.75 to −0.38) and an increase in pain resilience (effect size = 0.62) in both the groups when comparing the baseline to the 3-month follow-up. These improvements were maintained or further enhanced from post-intervention to the 3-month follow-up [32].

Furthermore, in individual case studies, Greenberg et al. [34] reported improvements in pain catastrophizing in all four patients and improvements in pain resilience in two out of the four patients. A study by Gmuca and colleagues [37] based on a PRISM program targeting resilience and coping skills in adolescents with chronic musculoskeletal pain showed an increase in self-perceived resilience after the intervention, while the levels of pain catastrophizing remaining similar before and after the intervention. Qualitatively, the authors reported that both the patients and the caregivers expressed a desire to continue focusing on building resilience through psychotherapy.

Finally, a study based on a modified Mindfulness-based Stress Reduction (MBSR) by Chen et al. [33] demonstrated its efficacy in reducing pain catastrophizing in adults with chronic pain (with effect size of −0.60) compared to that of a group with usual treatment, but this effect did not persist at 3-month follow-up. Moreover, regarding both the general and pain-related resilience levels, no significant differences were observed [33].

### 3.6. Physical Pain

The subjects with low back pain who participated in a pain education intervention reported a more significant reduction in both pain severity and pain interference from pre- to post-intervention [29] compared to that of the control group, which underwent a physiotherapy program and showed improvements only in measures of pain interference with daily life. The more structured 3RP intervention also observed a reduction in pain [35]. In the same way, the GetActive group with and without Fitbit clinical trials based on 3RP showed clinically significant reductions in pain levels during rest (effect size ranging from 0.95 to 1.30), and the GetActive group with Fitbit also showed this during activity (effect size of 1.12) [38]. Subsequent developments of this trial on a larger sample of participants (n = 82) confirmed clinically significant and medium-sized reductions in pain while at rest (effect size from 0.49 to 0.58) and during action (effect size from 0.59 to 0.70) [30]. Moreover, in the individual cases who underwent the GetActive-Fitbit program, Greenberg [34] reported an increase in the number of daily steps, an improvement in the distance walked in 6 min, in self-reported physical activity, and in physical function, variables that can be considered good markers of clinical outcomes and reflect a reduction in physical pain, in addition to a reduction in the pain levels at rest and during activity. In contrast with these observations, Basque and colleagues [36] reported a significant decrease in pain after treatment (effect size of 1.15); however, this was not maintained at the 3-month follow-up. In another study by Gmuca et al., no changes were found in the pain levels assessed from the baseline and immediately post-treatment, although improvements were found in the levels of functional disability, another pain-related clinical index [37]. Finally, in a study by Chen et al. [33], adult patients with chronic pain undergoing the modified MBSR program reported less pain interference at the end of treatment (effect size of −0.91) for up to 3 months of follow-up (effect size of −0.56) compared with that of the control group (treatment as usual). However, no differences in pain severity were found between the two groups.

### 3.7. Anxiety and Depression

Twenty subjects with low back pain who underwent pain psychoeducation reported less impairment in emotional functioning than that of the control group who underwent a physical therapy program [29]. In particular, the levels of depression in the experimental group appeared to be reduced by twice as much as those in the physical program group. Moreover, the 3RP program reported a decrease in the rates of depressive symptoms in patients with Neurofibromatosis [35]. These results were also supported by a reduction in anxiety levels in the same sample with effect sizes ranging from 0.73 to 1.33 [35]. On the other hand, a preliminary GetActive program also based on the 3RP program found non-significant improvements in anxiety and depression [38]. Subsequently, on larger samples, Greenberg and colleagues showed that the GetActive programs with Fitbit were associated with small-to-moderate effect sizes (from 0.40 to 0.54) for improvements in emotional function, including symptoms of anxiety and depression [30,34]. In another study, Grunberg et al. [31] observed the relationships between anxiety–depressive symptoms, pain catastrophizing, mindfulness skills, and resilience to pain. The authors found that the reduction in pain catastrophizing, followed by improvements in mindfulness and pain resilience after the intervention, accounted for much of the variance in the improvement of depressive symptoms. Furthermore, improvements in anxiety from baseline to post-treatment were primarily explained by reductions in pain catastrophizing and enhanced mindfulness skills, rather than by resilience. These findings suggest that targeting pain catastrophizing and mindfulness skills is crucial for improving emotional functioning in patients with chronic pain. One study also highlighted the effectiveness of a self-compassion intervention, reporting significant improvements in anxiety and depressive symptoms from pre- to post-treatment (effect sizes of 0.54 and 0.66, respectively), which were sustained at the 3-month follow-up [36]. On the other hand, in a study by Chen and colleagues [33], no significant differences in depressive symptoms were observed in either the experimental or control group after a modified MBSR intervention, while Gmuca and colleagues [37] reported an improvement in the levels of psychological distress, although not statistically significant in a resilience coaching program.

### 3.8. Other Psychological Outcomes

In addition to alleviating anxiety and depressive symptoms, interventions based on the principles of mind-body medicine have shown a positive impact on life satisfaction (effect size 0.73–1.33) in patients with chronic pain due to Neurofibromatosis [35]. These interventions were also associated with increased perseverance, balance, oneness, self-confidence, and a deeper search for meaning in life with effect sizes ranging from 0.73 to 1.33. Consistently with these results, the measures of post-traumatic awareness and growth also improved [35]. The authors also reported a reduction in the impact of stress (effect size 0.73–1.33) and the tendency to somatization (effect size of 0.54) after the intervention [35]. These data are confirmed by measures of quality of life, which showed a benefit after the intervention [29,38]. The quality of the sleep–wake cycle was also closely associated. When compared with the physiotherapy programs, the psychological pain interventions were helpful in reducing sleep disturbance [29,38], showing a trend toward decreased perceived sleepiness (effect size 0.92) [35].

Some authors used the FitBit in combination with the GetActive mind-body program and reported improvements in pain management, kinesiophobia (effect size −0.34–0.61), and coping skills (effect size 0.4–1.06) [30,32,34,38], as well as improved stress management skills, increased body awareness [38], and improved mindfulness skills (effect size of −0.44) [30,31,32,34], generally maintained at the 3-month follow-up in both the groups (with and without FitBit). Greenberg and colleagues [34] also showed improvements before and after treatment in emotional support, social isolation, and the impression of change. On the contrary, only one study reported no benefit in perceived social support [35]. Regarding the mindfulness skills, the modified MBSR program of Chen and colleagues [33] reported opposite results. In fact, they did not report efficacy in improving mindfulness skills in the experimental group, although they detected a larger reduction in perceived stress in the modified MBSR group compared with that of the group with usual treatment for chronic pain. This reduction was not detected at the end of the intervention, but only at the 3-month follow-up. In addition, this study was the only one based on the use of functional MRI, observing greater homogeneity in the anterior lobes of the cerebella in the participants subjected to modified MBSR, suggesting an influence of the intervention on brain plasticity, and in this specific case, on an area related to the regulation of muscle tension, one of the causes of pain and stiffness in movement [33]. Other studies have reported a significant increase in self-compassion, with an effect size of 0.92 [36], especially at the end of the intervention, but without significance at the follow-up after 3 months. Finally, Gmuca et al. [37], in their study based on resilience training, found no significant differences in overall health or the other indicators of psychological distress.

## 4. Discussion

This study reviewed the recent literature on the employment of psychological and psychotherapeutic interventions in patients suffering from chronic pain, particularly focusing on studies that included resilience and catastrophic thinking among the evaluated outcomes.

Ten studies including psychological and psychotherapeutic interventions in various clinical conditions characterized by chronic pain were considered. Identifying the psychological interventions that enhance the well-being of patients with persistent pain is crucial to complement the traditional medical therapies, which often fall short in effectively addressing such conditions. Overall, the psychological interventions demonstrated a beneficial impact on both the physical and mental health outcomes of the individuals living with chronic pain. In fact, the psychological interventions improved the patients’ pain perception, quality of life, emotional functioning, and perceived stress in the context of different diagnoses, such as rheumatologic pain, diabetes, cardiovascular problems, irritable bowel syndrome, and headaches [35,41], and in a group of patients with chronic temporomandibular joint disease [42]. The results of our study may suggest that different types of psychological interventions are effective in reducing pain catastrophizing [29,32,35,36] and increasing levels of resilience [32,35,36,37], despite some opposing, conflicting, or non-durable results [29,33,37].

The results regarding the reduction in pain levels after the interventions are mixed, as in some studies, a reduction in pain levels at rest and during activity was found [29,30,34,35,38], while in others, no results were found [33,37], or the improvements were not maintained at the follow-ups [36]. However, the study by Chen and colleagues [33] suggested that some types of psychological intervention such as MBSR might be effective not so much in reducing pain perception, but in improving patients’ pain management. Indeed, better mindfulness skills could foster patients’ ability to refocus attention on the present moment, promoting the more benign acceptance of pain. In fact, mindfulness was correlated with an improvement in pain response, and this effect was mediated by a reduction in catastrophizing [43]. The results observed on the measures of psychophysical well-being demonstrated the positive effect of applying a psychological intervention over the physical rehabilitation-based guidelines (e.g., physiotherapy treatment) [29]. Reducing the tendency to catastrophize might therefore require expanding the search for effective coping strategies, encouraging a more flexible view of stressful events. Also, increased levels of resilience could enable the greater adaptive capacity to cope with chronic pain. Indeed, it has been seen that resilience can attenuate the relationship between negative psychological processes and pain-related suffering, highlighting the role of resilience in pain adaptation [44]. It would be interesting to explore, in the context of physical pain and catastrophizing, the application of the existing theoretical models and interventions, such as Padesky and Mooney’s [45]. strength-based cognitive-behavioral therapy model. This is a four-step cognitive behavioral intervention oriented towards building a personalized resilience model, which has already shown effectiveness in the treatment of anxiety disorders and depression. As these disorders share negative, repetitive, and dysfunctional thinking as a common element, it is hypothesized that a resilience-centered intervention could help reduce catastrophic thinking. Specifically, Grunberg et al. [31] observed that pain catastrophizing, mindfulness, and pain resilience uniquely and fully functioned as the mediators of the level of depression from the baseline to post-treatment. Further, pain catastrophizing and mindfulness, but not pain resilience, uniquely and fully mediated the level of anxiety from the baseline to post-treatment. These findings highlighted that the reduction in catastrophizing and the increase in mindfulness and resilience skills could be crucial in determining the psychological health of patients with chronic pain. Since catastrophizing is considered as a cognitive bias [13], it could be treated in psychotherapy trials. Grunberg and colleagues [31] suggested that patients with chronic pain might benefit from addressing negative pain-related thoughts in psychotherapy with the help of different approaches that could improve pain management, such as through mindfulness interventions, resilience-focused interventions, catastrophizing, or pain acceptance. In general, the reviewed interventions emphasized normalizing pain as a common and shared experience across life stages, highlighting the weak correlation between pain and organic damage. This approach aimed to alleviate the negative concerns and expectations regarding illness or potential physical harm [29]. In this sense, reducing catastrophic thinking might help improve pain and stress management skills to foster the development of resilience [35]. Indeed, the fear avoidance model of pain by Crombez et al. [17] provides an important key to understanding the relationship between catastrophizing and resilience in the context of pain. Pain catastrophizing amplifies vigilance and fear, causing the individuals to avoid daily activities, limiting their interaction with the environment and restricting attention to threatening signals. This cognitive process reduces the individual ability to cope with pain in a flexible and adaptive manner, hindering the development and employment of resilience skills. Consequently, work on catastrophic beliefs could foster a more balanced and adaptive response to pain, promoting the choice of psychological resources that are functional in sustaining people’s psychophysical well-being in the long term. Psychological interventions have been shown to be effective in alleviating the burden of pain across various aspects of personal life, including daily activities, work, social relationships, sleep, and emotional well-being [29]. These findings are further supported by the larger improvements in quality of life [29,38], life satisfaction, mindfulness, post-traumatic growth, pain management, and stress reduction [30,32,33,34,38] experienced by patients undergoing psychological interventions, as compared to those receiving only physical treatments. In addition, some studies have explored the feasibility of the intervention [29,30,33], reporting high acceptability scores, good treatment adherence, and participant satisfaction. However, the absence of control groups in four studies limits the conclusions regarding the effectiveness of the examined interventions. Despite this, the positive feasibility and acceptance of psychological interventions among the study participants indicate promising prospects for the future. This suggests not only an increased openness to such approaches, but also the potential for a positive impact on pain reduction, mental health improvement, and their integration into clinical practice.

This systematic review did not include three studies exploring resilience [46,47]. These studies analyzed the resilience construct as multifactorial, a dynamic process in which stable personal resources (e.g., optimism and self-efficacy) may positively combine with factors that vary over time (positive emotions and social support). In fact, two studies did not consider resilience as a construct, but looked at it in terms of a personal strength [46,48], while one study included a resilience-based intervention, but did not measure it as an outcome of the intervention [47]. Research in line with this interpretation of the resilience construct, for example, has observed that resilient people use coping strategies that favor positive emotions [20]. For the purposes of this study, however, it was decided to include only those studies that considered resilience as a construct, evaluating it through psychometric instruments and as a clinical outcome of a psychological intervention.

Despite the promising results, our findings should be considered accounting for several limitations. First, the limited number of studies reviewed. There are a few studies investigating the application of intervention protocols to assess their impact on people’s psychological and physical well-being in relation to changes in resilience and catastrophizing skills. Future studies with larger samples could provide more robust and reliable evidence.

Second, the studies analyzed samples of patients with chronic pain attributable to different medical diagnoses. The different diagnoses could influence the effectiveness of interventions in distinct ways, which justifies the more specific categorization of patient types in future research. Consequently, the small number and inhomogeneity of the individuals treated could have affected the generalization of results. Third, some patients received medical treatment in parallel with psychotherapeutic interventions, which influenced the results on the effectiveness of psychological intervention alone. Fourth, it would be appropriate to differentiate the results on the treatment of anxiety and depression by checking the family and personal histories of psychiatric disorders. The presence of pre-existing psychiatric conditions could significantly influence the results. An assessment of family and personal mental health history would help to clarify whether the effects observed in some studies are due to the psychological interventions themselves or to the management of underlying psychiatric conditions. This differentiation would help provide more precise conclusions on the role of psychotherapeutic interventions in improving resilience and reducing catastrophic thinking [35].

Fifth, the lack of long-term follow-up results prevents drawing generalized conclusions about the effectiveness of these interventions over time.

Sixth, the psychotherapeutic interventions included in the study are heterogeneous in both content and duration. For example, some studies use Cognitive Behavioral Therapy (CBT), while the others employ mindfulness-based approaches or psychodynamic psychotherapy. Heterogeneity in the protocols could influence the effectiveness of psychological treatments. Future research should attempt to better define the characteristics of interventions to understand which therapeutic models are most effective for patients with chronic pain.

Seventh, most of the included studies were conducted in Western countries, such as the United States, where the cultural conceptions of mental health and pain might differ from those in other cultures. This may limit the generalization of results to populations in non-Western countries. Future studies should explore the effectiveness of psychological interventions in different cultural contexts to improve understanding of how such treatments may be adapted to different populations.

Eighth, in our systematic review, we decided to exclude non-English language studies. Although this decision may exclude relevant studies published in other languages, we chose to include only English language articles to ensure greater consistency in the selection and analysis of studies, considering that English is the predominant language in the international scientific literature. Furthermore, this choice facilitated the review process, data extraction, and the interpretation of results, as our team primarily has language skills in English. Including only English language studies is a common practice in systematic reviews to reduce the risk of interpretation errors related to translation and minimize potential bias. Moreover, as observed by Nussbaumer-Streit and colleagues [49], the exclusion of non-English publications does not have a significant impact on overall conclusions and may represent a useful methodological strategy, especially in reviews of clinical interventions.

Furthermore, a few studies have included the possibility of totally remote delivered interventions. In fact, the conditions of chronic pain may lead to the risk of rejection and abandonment because patients may have difficulties in joining in-person therapy [35]. Therefore, future studies could consider implementing remote interventions to overcome this limitation.

Finally, as mentioned above, the methodological limitations of the eligible studies, including the absence of control groups, the failure to control for confounding factors, drop-out and missing data rates, and the heterogeneity of research designs, compromised the possibility of drawing definitive conclusions on the effectiveness of the analyzed interventions. To overcome this limitation, a narrative synthesis was preferred to qualitatively describe the main findings. Future studies employing more rigorous and standardized methodologies are essential to validate these findings and provide more robust evidence on the effectiveness of the analyzed interventions.

## 5. Conclusions

The results of this systematic review provide a promising framework for integrating psychological and psychotherapeutic interventions into multidisciplinary treatment pathways for pain management in physical conditions. These interventions focus on enhancing resilience skills, reducing catastrophizing, and significantly influencing pain perception, along with the key aspects of patients’ psychophysical well-being, such as quality of life and personal satisfaction. Several factors might contribute to the sustained effectiveness of psychological interventions for pain management. Tailoring the interventions to the patient’s specific medical condition, involving patients in the goal-setting process, and adapting the interventions accordingly can enhance motivation and engagement, thereby promoting long-term outcomes. Regular follow-up, the reinforcement of coping strategies, and continued support throughout and after the intervention could help maintain the treatment benefits. Integrating psychological interventions within a multidisciplinary approach, alongside medical treatments, and addressing co-occurring psychological conditions may further support the durability of the results.

The findings may have several important clinical implications. The main clinical implications point to the integration of psychological and psychotherapeutic interventions within multidisciplinary therapeutic pathways for pain management, with a focus on the early identification of patients who might benefit from such interventions based on the levels of catastrophizing and resilience.

Firstly, the findings highlight the importance of integrating psychological interventions as a crucial component in pain treatment. Specifically, our results suggest that adopting personalized therapeutic strategies tailored to individual patients’ characteristics (such as the levels of catastrophizing and pre-existing resilience) could optimize the therapeutic outcomes. This personalized approach represents a significant advancement towards more patient-centered care, which can better address patients’ specific physical and emotional needs.

Second, on a preventive level, the promotion of resilience appears particularly promising. Helping patients to develop internal resources and coping strategies could not only improve the experience of pain, but also reduce the risk of future exacerbation and the onset of associated psychological disorders, such as anxiety or depression. 

Third, in many healthcare settings, the management of chronic pain primarily focuses on pharmacological treatments and surgical interventions. However, these approaches are not always sufficient and incur significant long-term costs. The integration of psychological interventions in pain treatment could reduce the dependence on pharmacological therapies, helping to maintain healthcare costs. Since psychological treatment aims to improve resilience and reduce catastrophic thinking, it could contribute to more sustainable long-term pain management, resulting in lower costs for healthcare systems related to analgesic drug use and hospital treatment. Most psychological interventions, however, require advanced skills that only licensed psychologists and psychotherapists possess. If these interventions are not carried out by trained experts, there is a risk of compromising their effectiveness, thus diminishing the long-term benefits for patients and the comparability of outcomes across interventions. A biopsychosocial approach could be appropriate to treating pain in a comprehensive and effective manner. Psychological interventions are crucial for addressing the psychological component of pain, enhancing emotional regulation, reducing dysfunctional thoughts, and promoting more adaptive coping strategies. While acknowledging the importance of psychologists’ involvement in pain treatment programs, it is equally vital that other healthcare professionals (such as physicians, nurses, and physical therapists) are sensitized and trained to recognize signs of psychological distress in patients and to facilitate access to psychological interventions. This training should include the ability to identify patients who may benefit from specific psychological interventions at an early stage, as well as acquiring basic psychological skills to support the emotional management of pain, thereby improving the overall experience of patients suffering from chronic pain. The adoption of such practical approaches could contribute to more personalized care and optimize therapeutic outcomes in the treatment of physical pain.

In conclusion, our findings suggest that the integration of psychological strategies in the treatment of physical pain may be promising. However, more research is needed to better evaluate the effectiveness of these interventions. Future studies should include larger samples, classify patients according to specific diagnoses, and differentiate between the effects of psychological and pharmacological interventions. Furthermore, the inclusion of long-term follow-ups and the analysis of more consistent intervention protocols could provide clearer indications. It is also important to explore the application of these interventions in different cultural contexts and through remote modalities in order to broaden their accessibility.

## Figures and Tables

**Table 1:** PIO framework.

Component	Inclusion Criteria	Exclusion Criteria
Population	Patients experiencing pain in any clinical or experimental context. No limits on age, gender or status	Patients who did not report physical pain condition.
Intervention	Psychological interventions in patients with physical pain.	Interventions not directly related to the psychological approach, such as medical, pharmacological, and physiotherapy interventions.
Outcomes	Measurement of catastrophic thinking and resilience as primary or secondary outcomes using validated tools.	Studies that did not measure catastrophic thinking or resilience as distinct constructs or only as a facet of other variables (e.g., positive emotions, self-efficacy, acceptance of pain or negative thinking, rumination or magnification).
Study Design	Randomized controlled trial; nonrandomized pilot trial; single group nonrandomized trial; single cases; all studies published in English.	Reviews, book chapters, editorials, conference abstracts and notes. Observational studies. Non-English studies.

Note: PIO **=** Population, Intervention, Outcome.

## Data Availability

Available on request.

## Appendix A

**Table A1:** Summary of Study Characteristics on Psychological Interventions.

Authors (Year of Publication, Country)	Study Design	Sample (n., Gender, Age)	Diagnosis	Psychological Intervention	Session Performed Number; Session Modality	Control Group	Variables Evaluated	Main Results
Sharma et al. [29] (Nepal)	RCT	Tot = 40 (M = 28; F = 12); Mean age = 41 y; IG n = 20 (M = 15; F = 5); CG n = 20 (M = 13; F = 7);	Non-specific Low Back Pain	Pain Education Intervention and home program	The intervention protocol lasted one hour and included a one-hour individual and in-person treatment session.	Guideline-based physiotherapy treatment	- Feasibility of the intervention - Therapeutic compliance - Level of satisfaction - Pain intensity - Pain interference - Sleep disturbance - Depression - Quality of life - Pain Catastrophizing - Resilience	Feasibility criteria were met IG: < pain intensity < pain interference < sleep disorder < depression < pain catastrophizing (more that CG) > quality of life No significant pre- and post-intervention difference in resilience CG: < pain interference < depression < pain catastrophizing No significant pre- and post-intervention difference in pain intensity, sleep disturbance, quality of life and resilience No difference in dropout rates, contamination, credibility, difficulty and satisfaction between groups.
Greenberg et al. [30] (USA)	RCT	Tot = 82 (M = 28; F = 54) 21–79 y; IG n =41; CG n =41;	Heterogenous musculoskeletal chronic pain	GetActive With Fitbit, a cognitive-behavioral oriented mind-body activity program based on the modified 3RP mind-body program for chronic pain in a group setting.	The final GetActive and GetActive-Fitbit programs include 10 weekly 90-min group sessions. The GetActive and GetActive-Fitbit programs are identical in content and structure. In the GetActive-Fitbit program, participants used a Fitbit device. The Fitbit component was the only difference between the programs.	Educational intervention	- Physical function - Physical activity - Depression - Anxiety - Pain at rest - Pain with activity - Pain resilience - Pain catastrophizing - Kinesiophobia - Adaptive coping - Mindfulness abilities	- Physical function - Physical activity - Depression - Anxiety - Pain at rest - Pain with activity - Pain resilience - Pain catastrophizing - Kinesiophobia - Adaptive coping - Mindfulness abilities
Grunberg et al. [31] (USA)	RCT	Tot = 82 (M = 28; F = 54) 21–79 y; IG n =41; CG n =41;	Heterogenous musculoskeletal chronic pain	GetActive With Fitbit, a cognitive-behavioral oriented mind-body activity program based on the modified 3RP mind-body program for chronic pain in a group setting.	The final GetActive and GetActive-Fitbit programs include 10 weekly 90-min group sessions. The GetActive and GetActive-Fitbit programs are identical in content and structure. In the GetActive-Fitbit program, participants used a Fitbit device. The Fitbit component was the only difference between the programs.	GetActive without Fitbit	- Anxiety - Depression - Pain - Mindfulness abilities - Pain Catastrophizing - Pain Resilience Scale	IG + CG: > depression from baseline to post-treatment was explained by pain catastrophizing, followed by mindfulness, and pain resilience > anxiety from baseline to post-treatment was explained by pain catastrophizing and mindfulness, but not by pain resilience.
Greenberg et al. [32] (USA)	RCT	Tot = 82 (M = 28; F = 54) 21–79 y; IG n =41; CG n =41;	Heterogenous musculoskeletal chronic pain	GetActive With Fitbit, a cognitive-behavioral oriented mind-body activity program based on the modified 3RP mind-body program for chronic pain in a group setting.	The final GetActive and GetActive-Fitbit programs include 10 weekly 90-min group sessions. The GetActive and GetActive-Fitbit programs are identical in content and structure. In the GetActive-Fitbit program, participants used a Fitbit device. The Fitbit component was the only difference between the programs.	GetActive without Fitbit	- Pain resilience - Pain catastrophizing - Kinesiophobia - Adaptive coping - Mindfulness abilities	Baseline to 3-Month Follow-up IG + CG: < pain catastrophizing < kinesiophobia > pain resilience > adaptive coping > mindfulness abilities Post-Intervention to 3-Month Follow-up IG + CG: Sustained or increased treatment gains on pain catastrophizing - pain resilience - mindfulness abilities - adaptive coping CG: > kinesiophobia
Greenberg et al. [34] (USA)	Single cases	Tot = 4 F	Heterogenous musculoskeletal chronic pain	GetActive With Fitbit, a cognitive-behavioral oriented mind-body activity program based on the modified 3RP mind-body program for chronic pain in a group setting.	The final GetActive and GetActive-Fitbit programs include 10 weekly 90-min group sessions. The GetActive and GetActive-Fitbit programs are identical in content and structure. In the GetActive-Fitbit program, participants used a Fitbit device. The Fitbit component was the only difference between the programs.	NA	- Physical Function - Levels of physical Disability - Physical Activity - Depression - Anxiety - Pain at rest - Pain during activity - Mindfulness abilities - Catastrophic thinking - Kinesiophobia - Coping skills - Social isolation - Emotional support - Pain resilience - Program satisfaction - Impression of Change	> number of daily steps > distance walked in 6-min > self-reported physical activity > physical function via PROMIS < disability > emotional function < depression < anxiety
Chen et al. [33] (China)	Pilot RCT	Tot = 67; IG n = 40 (M = 8; F = 32) mean age 32.92 CG n = 27 (M = 6; F = 21) mean age 30.89 y	Heterogenous musculoskeletal chronic pain	Modified 8-week Mindfulness-Based Stress Reduction Program (MBSR).	The modified MBSR included an 8-week group program. Each group met for about 2.5 h for weeks 1 and 8 and for 2 h per week for weeks 2–7. There was no one-day retreat practice required by the classical MBSR model. The total duration of the MBSR program was 17 h.	UC	- Pain severity - Pain interference - Depression - Perceived stress - Pain catastrophizing Mindfulness Abilities - Resilience - Differences in grey matter volume at functional MRI - Resting- state brain activity at functional MRI	IG: < pain catastrophizing, not maintained at T3 < pain interference < perceived stress > homogeneity in the anterior lobe of the cerebellum
Vranceanu et al. [35] (USA)	Single group nonrandomized pilot trial	Tot = 16 (M = 7; F = 9); Mean age = 57 y; NF1 n = 9 NF2 n = 6 Schwannomatosis n= 1	NF1, NF2 or Schwannomatosis	Relaxation Response Resiliency Program (3RP), a comprehensive outpatient program based on the principles of mind–body medicine.	The 3RP mind-body program featured 8 sessions at a weekly frequency of about 90 min. The meetings were held in a group setting of about 3–6 participants. The original intervention was modified so that it could be adapted to the specific needs of patients with NF1, NF2 and Schwannomatosis.	NA	- Satisfaction with life - Resilience (RS) - Perceived Stress - Mindfulness abilities - Optimism - Posttraumatic growth - Catastrophizing - Pain intensity - Daytime sleepiness - Eating behavior - Emotional support - Depression - Somatic Symptom Impact - Anxiety	> resilience > life satisfaction > mindfulness abilities > posttraumatic growth < stress < depression < anxiety < somatization < drowsiness < pain catastrophizing No significant differences in optimism, social support and eating behaviors.
Basque et al. [36] (Canada)	Single group nonrandomized pilot trial	Tot = 26 (M = 3; F = 23); Mean age= 57.5 y	General Chronic Pain	Self-compassion psychoeducation website	Self-compassion psychoeducation website was an individual 6-week program comprising exercises found on Neff’s psychoeducational website. Intervention comprised a video defining self-compassion, four self-compassionate writing exercises, guided meditation to be performed weekly and automated emails.	NA	- Self-Compassion - Pain Resilience Scale - Chronic Pain Acceptance - Pain Catastrophizing Scale - Illness Intrusiveness - Depression - Anxiety	Attrition higher Adherence to the protocol low Treatment satisfaction high > self-compassion > pain Resilience > chronic pain acceptance < pain < pain catastrophizing < anxiety < depression Most gains maintained or increased at follow-up
Gmuca et al. [37] (USA)	Single group nonrandomized pilot trial	Tot = 27 (M = 9; F = 18) 12–17 y; Median age= 15 y	Heterogenous musculoskeletal chronic pain	Promoting Resilience in Stress Management (PRISM), a cognitive-behavioral oriented resilience coaching program for youth with chronic illness.	PRISM is a resilience coaching program that involves four individual sessions of 30–50 min, administered about 1–2 weeks apart, for a total duration of about 3 months, followed by an optional fifth 30-min follow-up session. The mode of administration of the intervention was chosen by the patients: in person, via web communication or by telephone.	NA	- Quality of life - Resilience - Functional disability - Psychological distress - Pain catastrophizing - Pain intensity	Feasibility criteria were met High satisfaction < Functional disability > Self-perceived resilience < Psychological distress; No differences in Pain-catastrophizing, pain intensity, and quality of life pre- and post-intervention.
Greenberg et al. [38] (USA)	Nonrandomized pilot trial	Tot = 13 (M = 3; F = 10) IG n = 7; mean age= 41.6 CG n = 6; Mean age= 46.7	Heterogenous musculoskeletal chronic pain	GetActive With Fitbit, a cognitive-behavioral oriented mind-body activity program based on the modified 3RP mind-body program for chronic pain in a group setting.	The first version of the intervention included 8 weekly 90-min group sessions. The GetActive and GetActive-Fitbit programs are identical in content and structure. In the GetActive-Fitbit program, participants used a Fitbit device. The Fitbit component was the only difference between the programs.	GetActive without Fitbit	- Anxiety - Depression - Pain - Social Isolation - Emotional support - Mindfulness Abilities - Pain Catastrophizing - Kinesiophobia - Coping skills - Perceived Improvement	CG: < pain during rest and activity < pain resilience < kinesiophobia (non-significant), > coping (non-significant) IG: < pain during rest and activity > coping > pain resilience (non-significant). IG + CG: > anxiety (non-significant) > depression (non-significant) > physical function > number of steps

Legend: RCT = randomized clinical trial; IG = intervention group; CG = control group; UC = usual care; NA = not available; > = increase; < = decrease.
